# Gingivitis—Its Different Forms—Treatment, &c.

**Published:** 1878-04

**Authors:** M. E. Magitot


### ARTICLE II.

### *Gingivitis—Its Different Forms—Attempt to Classify its Varieties—Its Treatment by Monohydrated Chromic Acid.*

BY M. E. MAGITOT.

Translated from Le Progres Dentaire.

TRAUMATIC GINGIVITIS.—As we have remarked in our preceding classification, traumatic gingivitis comprises the following varieties:

1*st.* *Gingivitis of Smokers*, With carbon deposits.

2*nd.* *Tartaric Gingivitis*, Due to deposits of tartar at the border of the gum.

3*rd.* *Gingivitis of workmen in certain industries.*

Let us consider each of these successively:

1st. GINGIVITIS OF SMOKERS.—This sort of gingivitis consists essentially of inflammation of the free border of the gums with carbon deposits, blackish about the necks of the teeth. It is chiefly found in smokers who make an immoderate use of tobacco, and particularly in smokers who use cigarettes or pipes, both of which are of a nature to excite the greatest irritation in the buccal mucous membrane. It is that form of the disease that represents the first and most simple symptoms, ascribed by many writers to the use of tobacco, which beginning with simple gingivitis, develops into psoriasis and epithelioma in passing through the general stomatitis, forming aphthous patches and epithelial desquamation.

This form of gingivitis is often complicated with those we shall presently describe—that is, gingivitis due to tartaric deposits, which soon are followed with the carbon deposits, produced by the combustion of tobacco. This combination of the two varieties is essentially due to a more or less entire want of proper care of the individuals, who besides being smokers are negligent as to their teeth.

It follows from this that gingivitis is far from being of the same character in different smokers, for by taking some cleansing precautions they free themselves from its production. This gingivitis is then essentially due to direct irritation of the mucous membrane, caused by tobacco smoke, and to the deposits of carbon upon the free border, and as to this last circumstance let me say that it resembles a certain phenomenon pointed out by Gubler in people who use dentifrices of carbon, and which he designates as *lisere*, an embroidered border, of carbon.

Beyond these etiological characteristics which mark and differentiate gingivitis of smokers, the disease presents itself to our observation under the aspect of a red superficial band, comparatively small in height, and constituting an irregular inflammatory border. The free border of the gum is thickened into a roll, somewhat bare, without suppuration, strictly speaking, without heat or pain in the teeth, and these rolls rest in some manner upon another irregular border, adhering to the surface of the enamel and of the neck represented by the bed of carbon particles which cover them. The special appearance of the implicated gingivitis, in other respects, another peculiarity is, that it attacks only certain isolated points, such as the anterior portions of the superior and inferior dental arches, or in other words, the places most directly exposed to the irritating agents. The posterior portions are rarely affected.

These carbon layers ought to be carefully distinguished from other deposits which cover the surfaces of the teeth. They are invariably black, easily detached by means of a scraper, and composed of amorphous particles insoluble in all reagents. The other deposits are of a very different color: as for instance the greenish stains so common on the surface of the human teeth and which almost always cover the teeth of herbiferous animals. These last stains are formed of amorphous particles which furnish the coloring matter of vegetables. Various reagents dissolve them—the acids for instance. Besides these greenish stains, we will add the mass of muscosites and alimentary debris mixed with numerous parasites, which form more or less thick beds upon the teeth, of which the consistency and extent frequently augment in the course of acute diseases; thus then inaction of the mouth occurs from the suppression of the salivary secretions.

It is to these crust-like masses that writers in general, and M. Gubler in particular give the name, *mucus.* We have often objected to this nomenclature. In fact the mouth contains no traces of *mucus*, strictly speaking. The mucous membrane which carpets the oral cavity contains no traces of the muciparous glands; only the salivary glands pass a liquid product into the cavity, and it is the mixture of the liquids with the alimentary debris, and the epithelial layers in desquamation, the clusters of leptothrix, the parasites, etc., which constitute the deposits upon the surface of the necks of the teeth, whether combined or not with the mass of tartar.

Such is, in a few words, gingivitis of smokers: slight irritation, exclusion of the free border, and carbon deposits subjacent. We have nothing farther to say in a symptomatological point of view, of this form which is, in fine, one of the most superficial and one of the least serious of the various kinds of gingivitis.

2nd. Tartaric Gingivitis.—This form of gingival alteration is essentially the consequence of irritation produced by tartaric deposits, at times quite abundant, on the free border of the gum, which cover certain portions of the teeth and often the entire surface of the alveolar ridge. But we must here say a word or two concerning the nature and composition of tartar.

Tartar is a concrete stony mass, generally of a yellowish hue, capable of becoming *very* hard, and which is deposited on the surface of the teeth. The localities in which it is found are, by the order of their frequence—the posterior faces of the antero-inferior teeth, situated at the opening of the excretory ducts of the sub-maxillary and sub-lingual glands; the external faces of the superior molars in the vicinity of Steno's duct, and the inferior molars. It is rarely deposited on the lingual surface of the molars of either jaw, and is never found on the posterior face of antero-superior teeth which are not washed by saliva. These deposits of tartar which, when abundant, are indices of the constant alkaline action of the saliva, are often found in great abundance, when for some reason the teeth of one side take no part in mastication. In such case the deposits occur exclusively on the opposite. We have seen deposits of tartar so thick at times that they completely enveloped the teeth on all sides, in the mass. This phenomena is to be compared with the accounts of Pliny and other ancient writers, which tell of united teeth, constituting to all appearances, one single semi-circular tooth for each jaw.

Tartar is chiefly composed of mineral matter, phosphates and earthy carbonates, the relative proportion of which varies greatly according to different analyses. (Berselius, Vauquelin, Bibra.) Thus, at one time we find 60 per cent. of phosphates ; again, almost the same quantity of carbonates. We can very easily explain the difference in the results obtained ; thus, if the tartar analysed has been taken from superior molars, which are generally covered with deposits of parotidian saliva, there will be found a predominance of carbonates, as in the liquid itself. If it be taken from the posterior surfaces of inferior incisors it will be rich in phosphates. These variations of chemical composition are found in the constitution of salivary calculus and are explained in a like manner. The salts, carbonates or phosphates, are in the tartar mixed with and united to a certain quantity of organic matter—the epithelial cells, fatty globules, leucocytes, filiform algæ and infusoria of a species of *vibrio* and *monas*.

There have been several theories advanced upon the formation of tartar.

M. Serres has admitted the existence of tartaric glands located in the body of the gums and having the property of secreting the tartar of the teeth. Anatomical observations, however, have failed to demonstrate the existence of such glands.

Cl. Bernard's theory asserts that the formation of tartar is due to irritation of the alveolo-dental periostitis, as a consequence of denudation of the soft gums by alimentary fragments during the act of mastication. He compares this secretion to that which sometimes accompanies periostitis of the bone.

This explanation cannot be admitted, and unless we attribute dental periostitis to some secretory action, we need only to remark, in order to overthrow the doctrine that tartar is frequently deposited on bodies introduced into the mouth; such as artificial dentures in the complete absence of teeth and consequently of dental periostitis.

A third theory is that of M. Dumas, who assumes that there exists in the mouth two kinds of saliva, the one acid, the other alkaline, which supersaturates the first. The acid saliva holds the phosphates in solution, and when the alkaline saliva supersaturates the acid, the phosphates are precipitated.

This theory does not seem to us to conform exactly to truth. In our opinion, tartar is the result of a simple deposit, by precipitation, of phosphates and earthly carbonates held in solution in the saliva by virtue of the organic matter with which they are combined. Upon their arrival in the buccal cavity, the elements are decomposed in contact with the air and mucous membrane, the salts insoluble in water are precipitated and deposited on the surface of the teeth. This view is essentially that adopted by M. Gubler.

The quantity of tartar deposited in the mouth varies widely, according to the subjects and those differences are by our theory easily explained. In the one case, simple saliva may contain in certain individuals a less proportion of earthly salts in solution, and the tartaric deposit will be relatively slight; while in another the deposit may accidentally come into contact with an acid reagent which neutralizes it and causes it to enter into solution again in the saliva. If in the last case a deposit sufficiently small is found enveloped in very strong acid saliva, in spite of the neutralization of the tartar already formed, we can still observe an acid reaction at the necks of the teeth which often induces disastrous effects upon these organs.

The existence or absence of tartar in the mouth presents in the history of different diseases a certain signification, as follows: If very abundant, it indicates a free alkaline reaction of the saliva, like that in the centre where the teeth are situated, and if then it constantly excludes the caries of the teeth, it becomes the determining cause of gingivitis by simple traumatic irritation of the mucous membrane. On the other hand if there is but little tartar or none at all, it implies acid reaction of the saliva, and in the centre where the teeth are located, with all its consequences upon the condition of these teeth; then between these two extreme states, are grouped the various degrees of feeble alkaline acid or reaction and the various results produced by them.

Against this theory of the formation of tartar, there has been an objection urged on account of the great disproportion often observed of earthly phosphates which are so scarce in the saliva, whereas tartar contains about 60 per cent. This objection is without foundation, if we reflect that the quantity of saliva secreted, on an average, in forty-eight hours, in man is about 410 grammes, in which case, however little phosphates the salivary liquids contain, the formation of tartar can still be explained; for we know that this deposit is produced very slowly, and that many years are required to form a layer of much thickness.

The places chosen by the tartaric deposits, indicate directly the points which will be particularly effected with this kind of gingivitis; but we should further say that other accidental circumstances singularly favor the production of it; thus the loss of one or more teeth, certain diseases of the teeth, caries for instance, or at times an incomplete action, by simple negligence, of a whole side of the mouth. In case that the masticatory functions are performed only on the opposite side, the immediate consequence is that the active side free from all deposits is wholly normal, while the other, encumbered with calcareous masses is soon attacked with gingivitis.

We observe that the intensity of the gingival affection varies according to the quantity of tartar deposited.

In the most favorable cases, there is a simple reddish roll or band, raised by the tartar deposit, which has for its seat the interstice of the gingival border and dental surface, in other words the necks of the teeth. You very frequently find it on the anterior and posterior faces of the inferior alveolar border. The opposite region, the antero-superior, is nearly always free from it, as we have said above, on account of the absence of salivary secretion at this point, and also because the incessant motion of the tongue and lip continually cleanse the surfaces.

In a more strongly marked form, that is with a very abundant deposit of tartar, the affection is more extended. There is a festooned tumor with true absorption of the gums of the teeth, denuding them to a certain extent.

The gums then present a fungus appearance; they bleed at the slightest touch. Arrived at this stage, the disease is generally complicated with a certain degree of looseness and deviation in the teeth, due to the propagation of inflammation in the alveolo-dental periosteum. But as regards this, we have not extended our topic to the subject of periostitis itself, which only represents, after all, an after phenomenon and a consequence of primitive affection, and which must look elsewhere for its description.

To sum up, the essential and distinguishing characteristics of tartaric gingivitis are the results of the presence of tartar in deposits more or less extended and exclusively localized within the places of this material.

As to the treatment of this particular form, without wishing to here prejudge the rules of therapeutics which we will attempt to establish soon for gingivitis in general, we can say previously, that the application of these means will be invariably preceded by the simple removal of the deposits.

Further, you must attempt to cure the disposition or dental lesions that have been the cause of their production and apply hygienic agents with the view of chemically neutralizing the tartaric product on the place, or to check its return. This will be taken up in another lecture upon "*Rational Hygiene of the Mouth.*"

3rd. Gingivitis of Workmen in Special Industries.—With traumatic gingivitis, there are classed certain inflammations of the gingival mucous membrane, which are peculiar to some industries, independent of other lesions of the same order, which result from intoxication and which will be described with poisonous gingivitis.

In that class of gingivitis which has received the name of "*industrielle*," we find in the most conspicuous place that which was first described by Putegnat. He found it existing among the workmen of the glass factories of Baccarat, where 95 per cent. of the mechanics were affected to a greater or less extent.

The author from whom we borrowed this short description makes the remark—that the different conditions of temperature, of nourishment, of kinds of work, seem to exercise no influence upon the production of this disease; so that the time spent in the workshop seems to be the essen tial cause of its appearance.

The characteristics which it presents are: a tumefaction of the free border of the gums, with a swelling of the festoons along the margin of the dental interstices; the mucous membrane is red at first, then changes to a bluish black. It is a kind of *lisere* with thickening very different, according to Putegnat, from *lead lisere* which is in other respects in no way inflamed.

This distinction upon which the author carefully insists was necessary because the work of glass makers can, as we might suppose, lead to comparison with diseases due to leaden preparations.

Finally, there exists in this variety of gingivitis a local peculiarity very important for diagnosis, and one which, in some way, furnishes the etiological nature of it. It is that the disease is accompanied, or perhaps preceded, by caries at the necks of the teeth—caries that hasten to the rapid destruction of the crown, which as though a section was taken out at the level, breaks and falls in. The circumstance leads us directly to a peculiar consequence, viz: that we do not know how to compare gingivitis of glass workers to tartaric gingivitis, provided that the presence of tartar and dental caries exclude the usual reciprocity.

The lesion described by Putegnat is invariably located on the free margin of the gums without evidence of extension either to the buccal mucous membrane or dental periosteum. It follows from this that it is unaccompanied by loss or destruction of the teeth ; consequences quite frequent in the other numerous forms.

In the examination of the causes of this affection, Puteggnat makes allowances, it is true, for the surrounding circumstances, such as the humidity of the rooms, insufficient ventilation and other different common causes. But in his mind it is evident that the essential cause is the existence of fine glass dust in the factories, which fill the whole atmosphere and is deposited on the necks of the teeth, or that their presence excites the traumatic lesions with which they are affected.

Unable to produce here any personal experience, let us study again especially this kind of observation, and if we have, hereafter to explain the intervention here of the particles of dust in the production of concomitant caries, we shall easily succeed.

Pathogeny of gingivitis from the manufacture of glass can also be compared to many other analagous explanations ; thus it is an action of the same sort as produces ophthalmia in quarrymen, coal men, and it is also proper to recall here that the workmen or miners, as also the workmen of Baccarat, are affected with a disease much more serious and which results from the presence of foreign substances ; we refer to deposits of matter in the respiratory ducts that invite tuberculization in rapid effect.

However it may be, the gingivitis of glass-blowers is not the only disease met with in the industries ; as for example, workmen employed in the manufacture of arsenical preparations; manufacturers of colored papers, (Imbert Gourbeyre;) the manufacturers of iodic and phosphoric preparations, (Lebert et Wyss;) the mines of Almaden, (Roussel;) but these are forces considered mostly under the head of poisonous gingivitis, and which will be described later. The gingivitis of the manufacturers of Baccarat, is, it is true, the only one which has been so far clearly and nosologically described. But there are surely many others that are essentially of the traumatic character.—*Mo. Dent. Journal.*

